# Patient‐reported outcomes provide prognostic information for survival in patients with diffuse large B‐cell lymphoma: Analysis of 1239 patients from the GOYA study

**DOI:** 10.1002/cam4.4692

**Published:** 2022-03-23

**Authors:** Huang Huang, Asim Datye, Ming Fan, Andrea Knapp, Tina Nielsen, Alessia Bottos, Joseph N. Paulson, Peter C. Trask, Fabio Efficace

**Affiliations:** ^1^ F. Hoffmann‐La Roche Ltd Mississauga Ontario Canada; ^2^ F. Hoffmann‐La Roche Ltd Basel Switzerland; ^3^ Genentech, Inc. South San Francisco California USA; ^4^ Data Center and Health Outcomes Research Unit Italian Group for Adult Hematologic Diseases (GIMEMA) Rome Italy

**Keywords:** diffuse large B‐cell lymphoma, International Prognostic Index, patient‐reported outcomes, prognostic factor, survival

## Abstract

**Purpose:**

We investigated the prognostic value of pretreatment patient‐reported outcomes (PROs) in patients with diffuse large B‐cell lymphoma (DLBCL) receiving obinutuzumab/rituximab plus chemotherapy in the GOYA phase III study.

**Methods:**

Patients completed the European Organization for Research and Treatment of Cancer Quality of Life (EORTC QLQ‐C30) and the Functional assessment of chronic illness therapy‐Lymphoma (FACT–Lym) lymphoma subscale (LYMS) during the study. PRO scales with high prognostic value were identified through Cox regression analyses of overall survival (OS) and progression‐free survival (PFS). These scales were evaluated in terms of their additional prognostic value beyond the International Prognostic Index (IPI). A preliminary assessment was performed to evaluate whether the scales provided improved patient‐risk stratification beyond IPI.

**Results:**

One thousand two hundred and fifty‐nine patients with valid pretreatment PRO scales were included in the analyses, and complete pretreatment data were available for 1239/1414 patients (87.6%). Four PRO scales with high prognostic value were identified: FACT–Lym LYMS and EORTC QLQ‐C30 physical functioning, global health status/quality of life (QoL), and fatigue. All four scales retained significant prognostic value for OS and PFS after IPI adjustment (all *p* < 0.05). After adjusting for multiple clinical variables (IPI, cell of origin, *BCL2* status, and total metabolic tumor volume), all four scales retained significant prognostic value (all *p* < 0.05) for OS. Only the EORTC QLQ‐C30 physical functioning scale was significant (*p* < 0.05) for PFS after adjustment for multiple clinical variables.

**Conclusions:**

In this large population of patients with DLBCL, pretreatment PROs provided prognostic information for OS and PFS beyond the well‐established IPI.

## Graphical abstract

Patient reported outcomes were exhaustively reviewed for prognostic signal in GOYA ‐ the largest trial to date for DLBCL ‐ and multiple scales at baseline were prognostic even after multiple testing correction. The prognostic value was often orthogonal and additionally prognostic on top of the International Prognostic Index (IPI) clinical score used in DLBCL.

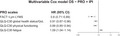

## 1 INTRODUCTION

Diffuse large B‐cell lymphoma (DLBCL) is an aggressive form of non‐Hodgkin lymphoma composed of biologically heterogeneous subgroups with variable prognosis.^1^ With modern immunochemotherapy, comprising rituximab, cyclophosphamide, doxorubicin, vincristine, and prednisone (R‐CHOP), approximately 60% of patients are alive at 5 years and considered cured.^2^ However, for patients who do not experience a response, or relapse early following first‐line treatment with R‐CHOP, outcomes are poor.^1^ Therefore, the optimal management of DLBCL remains an ongoing challenge, and the identification of suitable methods to improve and refine the prognostication of this biologically diverse disease remains a high priority.

The International Prognostic Index (IPI) has been widely used for more than 25 years as a prognostic model of outcomes in DLBCL.^3^, ^4^ The model incorporates several clinical and demographic parameters, including age at diagnosis, Ann Arbor stage, lactate dehydrogenase level, number of extranodal sites, and Eastern Cooperative Oncology Group performance status (ECOG PS), to estimate patient survival and individualize treatment.

Following the adoption of targeted immunochemotherapy as standard of care for DLBCL, the IPI was re‐evaluated in patients treated with rituximab‐based therapy and shown to retain its prognostic utility.^5^, ^6^

Over the last two decades, a number of oncology studies have also shown that patient‐reported outcomes (PROs), including functional aspects and symptoms, provide independent prognostic information for overall survival (OS). However, such evidence mainly stems from patients diagnosed with solid tumors, and data are scarce for patients with hematologic malignancies.^7^, ^8^, ^9^, ^10^, ^11^, ^12^ Even less information is available for DLBCL, with the notable exception of Jung et al.,^13^ who found an independent association between worse pretreatment PROs and survival in a sample of 263 patients treated with R‐CHOP.

GOYA was a randomized, phase III study assessing the efficacy and safety of obinutuzumab, a fully humanized, glycoengineered, type II anti‐CD20 monoclonal antibody, plus chemotherapy (G‐CHOP) versus R‐CHOP for first‐line DLBCL treatment.^14^ Final analysis of data from this study showed no significant difference in progression‐free survival (PFS) or OS for G‐CHOP versus R‐CHOP.^15^ Using data from the GOYA trial, the main objective of this analysis was to investigate the pretreatment prognostic value of PROs for OS and PFS beyond that of the well‐established IPI, in patients with DLBCL receiving first‐line immunochemotherapy. A secondary objective was to examine the prognostic value of PROs by disease risk (lower vs. higher) according to IPI score at diagnosis.

## 2 METHODS

### 2.1 Study design and patient population

The full GOYA study design has been described previously (Supplementary Material).^14^ Eligible patients were aged ≥18 years with previously untreated, histologically documented, CD20‐positive DLBCL. The primary endpoint was investigator‐assessed PFS, with OS as a secondary endpoint.

The GOYA study was conducted in accordance with the Declaration of Helsinki and the International Conference on Harmonization guidelines for Good Clinical Practice (GCP). The protocol was approved by the ethics committees of participating centers and registered at ClinicalTrials.gov (NCT01287741; https://clinicaltrials.gov/ct2/show/NCT01287741). All patients provided written informed consent.

### 2.2 PRO assessments

Patients completed the European Organization for Research and Treatment of Cancer Quality of Life (EORTC QLQ‐C30) questionnaire and the Functional assessment of chronic illness therapy‐Lymphoma (FACT–Lym) lymphoma subscale (LYMS) prior to treatment initiation and at regular intervals during the study.

The EORTC QLQ‐C30 consists of 30 questions, 24 of which are included in nine multi‐item scales.^16^ The multi‐item scales consist of five functioning scales (physical, role, cognitive, emotional, and social), three symptom scales (fatigue, pain, and nausea/vomiting), and one global health status/quality of life (QoL) scale. The remaining six single‐items assess the following symptoms: dyspnea, appetite loss, sleep disturbance, constipation, diarrhea, and financial difficulties. All scales and single‐items are transformed into standardized scores ranging from 0 to 100. A higher score for the functioning scales and global health status/QoL denotes a better level of functioning (i.e., a better state of the patient), while higher scores on the symptoms indicate greater symptom severity.

The FACT–Lym LYMS is a 15‐item subscale of the 42‐item FACT–Lym questionnaire, with each item scored on a 5‐point scale, ranging from 0 = “not at all” to 4 = “very much”.^17^, ^18^, ^19^ The FACT–Lym LYMS (range 0–60) assesses patient concerns relating to lymphoma and comprises common lymphoma disease and/or treatment‐related symptoms (e.g., pain, fever, swelling, night sweats, insomnia, itching, weight loss, fatigue, and loss of appetite). A higher score reflects better health‐related QoL.

### 2.3 Statistical analysis

As there was no statistically significant difference between treatments (G‐CHOP vs. R‐ CHOP) in terms of survival outcomes in GOYA,^14^, ^15^ data from both treatment arms were combined in these analyses to increase statistical power (i.e., more events for PFS and OS analyses). However, as a precaution all Cox proportional hazard (PH) regression analyses were stratified by treatment arms.

Univariate Cox regression analyses were performed for the FACT–Lym LYMS and pretreatment (i.e., baseline) scores from the 15 EORTC QLQ‐C30 scales to evaluate the prognostic value of these questionnaires in terms of PFS and OS. A complete case analysis was performed (i.e., patients with valid scores for the relevant pretreatment scale were included in each model). Demographic characteristics were similar for patients included versus excluded from the models (Table 1). The scales were ranked based on hazard ratios (HRs) and the respective 95% confidence intervals (CIs). The four highest‐ranking scales for both PFS and OS results were evaluated further, with HRs presented for 10‐point changes for descriptive purposes.

**TABLE 1:** Baseline demographic and clinical characteristics of patients from the GOYA dataset

Characteristic^a^	Patients without missing scale values^b^ (*n* = 1239)	Patients with ≥1 missing scale value^c^ (*n* = 175)	All patients (*n* = 1414)
Median (SD) age, years	61.0 (18.0–86.0)	65.0 (26.0–83.0)	62.0 (18.0–86.0)
Male	652 (52.6)	98 (56.0)	750 (53.0)
Bone marrow involvement			
Positive	132 (10.7)	21 (12.0)	153 (10.8)
Negative	1084 (87.5)	150 (85.7)	1234 (87.3)
Indeterminate	13 (1.0)	1 (0.6)	14 (1.0)
Missing	10 (0.8)	3 (1.7)	13 (0.9)
*BCL2* mutational status			
Positive	311 (25.1)	52 (29.7)	363 (25.7)
Negative	340 (27.4)	52 (29.7)	392 (27.7)
Missing	588 (47.5)	71 (40.6)	659 (46.6)
Cell of origin			
ABC	217 (17.5)	26 (14.9)	243 (17.2)
GCB	464 (37.4)	76 (43.4)	540 (38.2)
Unclassified	130 (10.5)	20 (11.4)	150 (10.6)
Missing	428 (34.5)	53 (30.3)	481 (34.0)
TMTV			
Median (range)	337 (1.04–17,100)	453 (2.18–7810)	353 (1.04–17,100)
Missing	83 (6.7)	15 (8.6)	98 (6.9)
ECOG PS			
0–1	1087 (87.7)	142 (81.1)	1229 (86.9)
2–3	151 (12.2)	33 (18.9)	184 (13.0)
Missing	1 (0.1)	0 (0)	1 (0.1)
Ann Arbor stage			
I–II	303 (24.5)	37 (21.1)	340 (24.0)
III–IV	936 (75.5)	137 (78.3)	1073 (75.9)
Missing	0 (0)	1 (0.6)	1 (0.1)
LDH			
≤280 units/L	605 (48.8)	61 (34.9)	666 (47.1)
>280 units/L	630 (50.8)	101 (57.7)	731 (51.7)
Missing	4 (0.3)	13 (7.4)	17 (1.2)
Extranodal sites			
0–1	814 (65.7)	98 (56.0)	912 (64.5)
2–3	347 (28.0)	67 (38.3)	414 (29.3)
4	39 (3.1)	5 (2.9)	44 (3.1)
>4	39 (3.1)	5 (2.9)	44 (3.1)
IPI score			
Low/low‐intermediate	702 (56.7)	79 (45.1)	781 (55.2)
High/high‐intermediate	537 (43.3)	95 (54.3)	632 (44.7)
Missing	0 (0)	1 (0.6)	1 (0.1)

Abbreviations: ABC, activated B cell‐like (subgroup); ECOG PS, Eastern Cooperative Oncology Group Performance Status; FACT–Lym LYMS, Functional assessment of chronic illness therapy‐Lymphoma lymphoma‐specific subscale; GCB, germinal‐center B cell‐like (subgroup); IPI, International prognostic index; LDH, lactate dehydrogenase; QoL, quality of life; SD, standard deviation; TMTV, total metabolic tumor volume.

^a^ All values are *n* (%) unless otherwise stated.

^b^ Patients with no missing values for any of the four scales: FACT–Lym LYMS, physical functioning, fatigue, and global health status/QoL.

^c^ Patients with missing values for at least one of the four scales: FACT–Lym LYMS, physical functioning, fatigue, and global health status/QoL.

In all subsequent analyses, for consistency (i.e., same sample size for any scale), only patients with valid pretreatment scores on all selected scales were included. A sensitivity analysis was performed to evaluate whether patients missing one or more of the selected scales (i.e., patients excluded from these analyses) would have significant differences in PFS and OS compared with patients included in subsequent analyses.

The added prognostic value to IPI for each of the selected scales was evaluated using Cox PH models adjusted for IPI expressed as two categories (high/high‐intermediate [H/HI]: 3–5, low/low‐intermediate [L/LI]: 0–2). The added prognostic value of each selected PRO scale modeled as continuous predictors was also assessed using the likelihood‐ratio test and calculation of the concordance index (C‐index). The likelihood‐ratio test evaluated whether adding a PRO scale to a Cox model adjusted for IPI provided significant value. The C‐index is defined as the proportion of all comparable pairs in which the predictions and outcomes are concordant, with 1 indicating perfect prediction accuracy and 0.5 indicating that the model is only as good as random prediction. A positive difference in C‐index between a model with a PRO scale and a model without would indicate additional prognostic contribution of the scale.

To assess the applicability of the PRO scales' added prognostic values as a proof‐of‐ concept, we evaluated the contribution of each scale to patient‐risk stratification in addition to IPI. Patients were grouped based on their respective IPI risk category (H/HI or L/LI), and the selected scales were dichotomized to indicate high and low PROs, based on their respective median score. Within each of the two IPI risk categories (stratified by treatment), patients with high PROs were compared with patients with low PROs using multivariable Cox regression. A statistically significant HR would indicate an important contribution of the scale to patient‐risk stratification for the specific IPI risk category.

As a sensitivity analysis, a multivariable model was used adjusting for IPI and known prognostic factors, including cell of origin (activated B cell‐like [subgroup], germinal‐center B cell‐like [subgroup], unclassified, and missing), *BCL2* mutation status (positive, negative, and missing), and total metabolic tumor volume (continuous).^20^, ^21^ Cell of origin was assessed using a gene‐expression profiling assay (Nanostring Lymphoma Subtyping Research Use Only Assay). TMTV was centrally determined by three experienced nuclear medicine physicians from each baseline PET/CT scan collected in real time during the study. TMTV was calculated using a tumor threshold of 1.5 times the mean SUV of the liver +2 standard deviations. Only those tumors that measured >1 ml were included in the TMTV calculation. In the GOYA study, patients with significant comorbidities were excluded. Therefore, comorbidities were not included in this sensitivity analysis. As an alternative, an exploratory analysis was performed that included the total number of comorbidities in the multivariable model.

Given the exploratory nature of the study, we did not control for type I error due to multiple testing, and an independent alpha level of 0.05 was assumed for each analysis. Statistical analyses were performed using R software version 3.6.3.^22^

## 3 RESULTS

### 3.1 Patient characteristics

In total, 1418 patients with previously untreated DLBCL were included in the intent‐to‐treat population in the GOYA study. At the time of the final data cutoff (January 31, 2018), four patients were excluded from the analysis due to GCP noncompliance at a single study site. Of the remaining 1414 patients, 1259 with at least one valid pretreatment PRO scale were included in initial screening that identified the following scales for further investigation of prognostic value: FACT–Lym LYMS and EORTC QLQ‐C30 physical functioning, global health status/QoL, and fatigue (Table 1 and Figure S1). A total of 1239 patients (87.6%) completed all four of the selected PRO scales before starting treatment, and key clinical characteristics were similar for patients with or without pretreatment PRO data (Table 1). Sensitivity analyses indicated no statistically significant difference in OS or PFS between patients with complete data and those with missing values for at least one of the selected PRO scales (Figure S2).

For the 1239 patients with complete PRO data, median patient age at diagnosis was 61 years and 52.6% of patients were male. Few patients (10.7%) had bone marrow involvement and the majority (87.7%) had an ECOG PS of 0/1. IPI score was categorized as L/LI for 56.7% of patients and as H/HI for 43.3% of patients.

### 3.2 Prognostic value of PRO scales for OS

**FIGURE 1:**
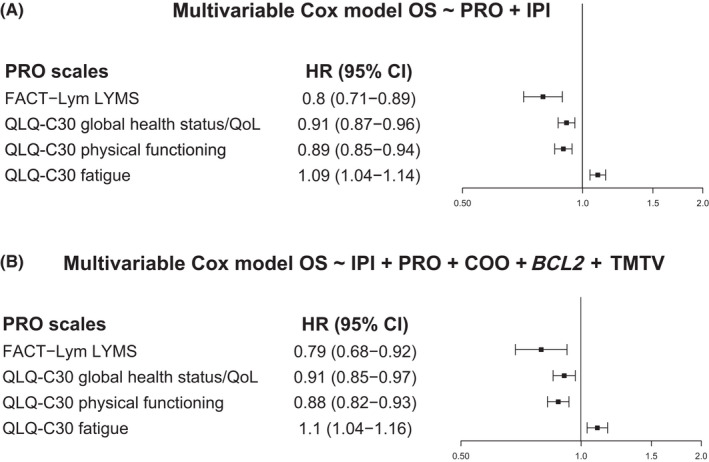
OS prognostic value of the four PRO scales. Cox regression analysis after adjustment for: (A) IPI* and (B) IPI, cell of origin, *BCL2*, and total metabolic tumor volume^†^. All four PRO scales were statistically significant in both Cox models. Higher scores for all four scales except the QLQ‐C30 fatigue scale indicate better QoL or functioning, while higher scores on the QLQ‐C30 fatigue scale indicate greater symptom severity. *Adjustment for IPI (high/high‐intermediate and low/low‐intermediate). ^†^Adjustment for IPI (high/high‐intermediate and low/low‐intermediate), COO (ABC, GCB, unclassified, and missing), *BCL2* status (positive, negative, and missing), and TMTV (continuous, mean imputed for missing). ABC, activated B cell‐like (subgroup); CI, confidence interval; COO, cell of origin; FACT–Lym LYMS, functional assessment of chronic illness therapy‐Lymphoma lymphoma‐specific subscale; GCB, germinal‐center B cell‐like (subgroup); HR, hazard ratio; IPI, International prognostic index; OS, overall survival; PRO, patient‐reported outcome; QLQ‐C30, European Organization for Research and Treatment of Cancer Quality of Life, Core 30; QoL, quality of life; TMTV, total metabolic tumor volume

Among the 1239 patients, 258 deaths were observed. The main causes of deaths were progressive disease (63%) and adverse events (28%). In the analysis for OS, all four scales selected for further analysis had prognostic value for OS after adjusting for IPI alone (Figure 1A), and after adjusting for IPI and other key variables (cell of origin, *BCL2* mutation status, and total metabolic tumor volume) in addition to IPI (Figure 1B). The FACT–Lym LYMS scale had the largest impact for OS (adjusted for IPI alone: HR, 0.80; 95% CI: 0.71–0.89; adjusted for IPI and other variables: HR, 0.79; 95% CI: 0.68–0.92).

### 3.3 Prognostic value of PRO scales for PFS

**FIGURE 2:**
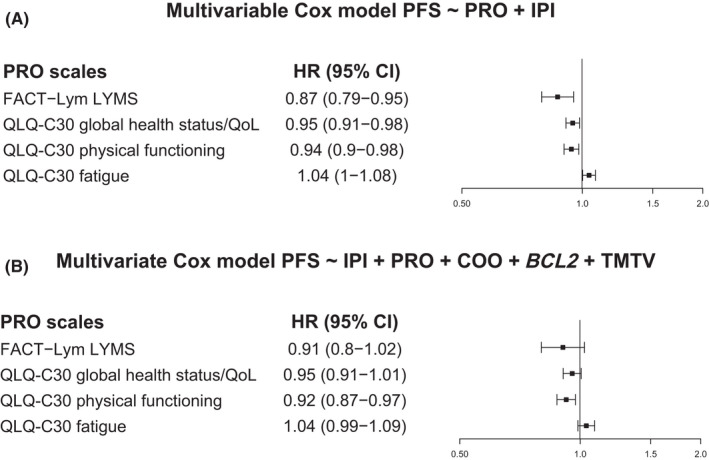
PFS prognostic value of the four PRO scales. Cox regression analysis after adjustment for: (A) IPI* and (B) IPI, cell of origin, *BCL2*, and total metabolic tumor volume^†^. After adjustment for IPI, the FACT–Lym LYMS and QLQ‐C30 global health status/QoL and physical functioning scales were statistically significant. After adjustment for IPI plus COO, *BCL2* status, and TMTV, the QLQ‐C30 physical functioning scale was statistically significant. Higher scores for all four scales except the QLQ‐C30 fatigue scale indicate better QoL or functioning, while higher scores on the QLQ‐C30 fatigue scale indicate greater symptom severity. *Adjustment for IPI (high/high‐intermediate and low/low‐intermediate). ^†^Adjustment for IPI (high/high‐intermediate and low/low‐intermediate), COO (ABC, GCB, unclassified, and missing), *BCL2* status (positive, negative, and missing), and TMTV (continuous, mean imputed for missing). ABC, activated B cell‐like (subgroup); CI, confidence interval; FACT–Lym LYMS, Functional assessment of chronic illness therapy‐Lymphoma lymphoma‐specific subscale; GCB, germinal‐center B cell‐like (subgroup); HR, hazard ratio; IPI, International prognostic index; PFS, progression‐free survival; PRO, patient‐reported outcome; QLQ‐C30, European Organization for Research and Treatment of Cancer Quality of Life, Core 30; QoL, quality of life; TMTV, total metabolic tumor volume

In the analysis for PFS, all four scales had prognostic value after adjusting for IPI alone (Figure 2A); only physical functioning was prognostic after adjusting for IPI and other variables (cell of origin, *BCL2* status, and total metabolic tumor volume) (Figure 2B). Of note, patients with a high baseline QoL had numerically smaller median TMTV versus patients with a low baseline QoL (Figure S4).

The median number of comorbidities per patient is 2 (range: 0–50). The most common comorbidities were hypertension (34%), diabetes mellitus (8.9%), and constipation (8.6%). None of them were found to be significantly associated with PFS or OS (Table S1). The prognostic values of all four PRO scales remained consistent after adding the number of comorbidities in the multivariable model (Figure S3).

### 3.4 Added prognostic value of PRO scales to IPI—for OS and PFS

All four PRO scales selected for further analysis (FACT–Lym LYMS and EORTC QLQ‐C30 physical functioning, global health status/QoL and fatigue) were found to have prognostic value for OS after adjustment for IPI, based on the likelihood‐ratio test, with each scale increasing the C‐index compared with the null model (no PRO scale included) (Table 2).

**TABLE 2:** Comparison of the predictive power for survival of the four PRO scales after adjustment for IPI

	*p*‐value for likelihood‐ratio test	Null model^a^ concordance index	Full model^b^ concordance index	Change in concordance index
OS				
IPI + FACT–Lym LYMS	<0.001	0.582	0.616	0.034
IPI + QLQ‐C30 physical functioning	<0.001	0.585	0.622	0.037
IPI + QLQ‐C30 global health status/QoL	<0.001	0.585	0.620	0.035
IPI + QLQ‐C30 fatigue	<0.001	0.586	0.619	0.033
PFS				
IPI + FACT–Lym LYMS	<0.01	0.571	0.593	0.022
IPI + QLQ‐C30 physical functioning	<0.01	0.573	0.592	0.019
IPI + QLQ‐C30 global health status/QoL	<0.01	0.573	0.597	0.024
IPI + QLQ‐C30 fatigue	<0.05	0.574	0.591	0.017

*Note*: Higher concordance index was associated with better performance of the PRO scale.

Abbreviations: FACT–Lym LYMS, Functional assessment of chronic illness therapy‐Lymphoma lymphoma‐specific subscale; IPI, International prognostic Index; OS, overall survival; PFS, progression‐free survival; PRO, patient‐reported outcome; QLQ‐C30, European Organization for Research and Treatment of Cancer Quality of Life, Core 30; QoL, quality of life.

^a^ Model without PRO scale.

^b^ Model with PRO scale.

Changes in C‐index ranged from 0.033 to 0.037, with the greatest increase shown for QLQ‐ C30 physical functioning scale (0.037).

All four PRO scales showed significant prognostic value for PFS after adjustment for IPI based on the likelihood‐ratio test (Table 2). Compared with the null model, the addition of each of these scales resulted in an increase in C‐index, with the largest increase observed for EORTC QLQ‐C30 global health status/QoL scale (0.024).

### 3.5 Prognostic value of PROs by disease risk (lower vs. higher) at diagnosis

**FIGURE 3:**
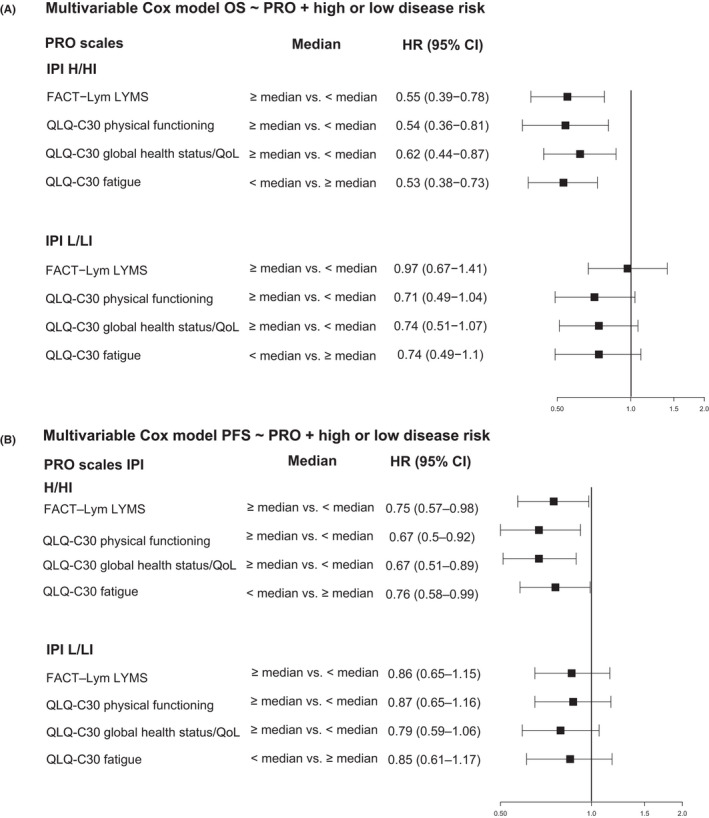
Cox model analyses of (A) OS and (B) PFS and PRO scales for patients with low versus high disease risk at diagnosis (stratified by treatment). All four PRO scales were statistically significant for PFS and OS in the IPI H/HI group. CI, confidence interval; FACT–Lym LYMS, Functional assessment of chronic illness therapy‐Lymphoma lymphoma‐specific subscale; H/HI, high/high‐intermediate; HR, hazard ratio; IPI, International prognostic index; L/LI, low/low‐intermediate; OS, overall survival; PFS, progression‐free survival; PROs, patient‐reported outcomes; QLQ‐C30, European Organization for Research and Treatment of Cancer Quality of Life, Core 30; QoL, quality of life

**FIGURE 4:**
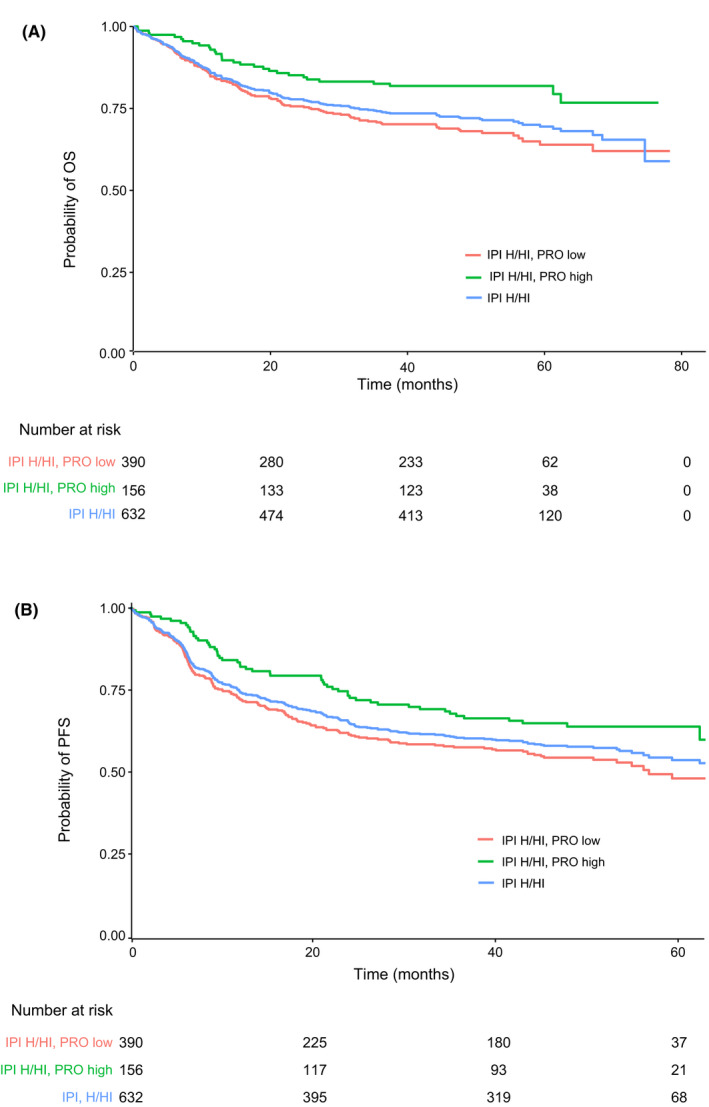
Kaplan–Meier analyses for (A) OS and (B) PFS among patients in the high‐risk IPI (H/HI score) group, by pretreatment patient‐reported QLQ‐C30 physical functioning score (low vs. high). PRO high refers to patients with a physical functioning score above the median. PRO low refers to patients with a physical functioning score below or equal to the median

The prognostic value of the four dichotomized PRO scales was evaluated within each of the two IPI groups, to determine whether the PROs added further prognostic information by differentiating a high‐risk group of patients. Each of the PRO scales had a significant association with both PFS and OS in the high‐risk IPI (H/HI score) group (Figure 3A,B). In contrast, none of the scales had prognostic value for either survival outcome in the low‐risk IPI (L/LI score) group. For descriptive purposes only, OS and PFS curves by pretreatment patient‐reported EORTC QLQ‐C30 physical functioning scores are depicted in Figure 4A,B, respectively. Median OS in the IPI high‐risk group was not evaluable (NE) for patients with low or high EORTC QLQ‐C30 physical functioning scores at baseline [both 95% CIs: NE–NE] and for all patients [95% CI: 74.6–NE]. Median PFS in the IPI high‐risk group was 56.8 months [95% CI: 45–NE] for patients with low baseline physical functioning scores, NE [95% CI: NE–NE] for patients with high baseline physical functioning scores and 66.0 months [95% CI: 56.8–NE] for all patients.

## 4 DISCUSSION

In this investigation of the prognostic value of PROs in 1239 patients with DLBCL receiving first‐line immunochemotherapy, we found that pretreatment PROs were prognostic for both OS and PFS. The FACT–Lym LYMS and QLQ‐C30 physical functioning, global health status/QoL, and fatigue scales were the highest‐ranking PRO scales in terms of prognostic value. The prognostic importance of pretreatment PROs remained for both OS and PFS even after adjusting for IPI score, and other key variables such as cell of origin, *BCL2* mutation status, and total metabolic tumor volume. This was most consistent for OS where all four scales remained significant in multivariable testing, whereas for PFS only the QLQ‐ C30 physical functioning scale remained significant when considering the IPI score plus other key potential prognostic variables. Across all four scales, FACT–Lym LYMS exhibited the strongest HR estimate in most analyses adjusting for IPI alone, followed by EORTC QLQ‐C30 physical functioning. However, only the EORTC QLQ‐C30 physical functioning scale was prognostic for PFS after adjusting for IPI plus other key variables. Also, the addition of this scale provided the largest increase in the C‐index (compared to other PRO scales) when considering the prognostic value of the IPI for OS. Based on these findings, we suggest that the EORTC QLQ‐C30 physical functioning scale should be regarded as a key PRO domain for clinical consideration when assessing patient risk in future DLBCL studies.

We found that PRO scales had more prognostic power in patients with high‐ versus low‐risk disease. Each of the PRO scales had a significant association with both PFS and OS in the H/HI IPI group, but were not associated with PFS or OS in the L/LI group. This finding may be corroborated by previous systematic reviews indicating that an association between PROs and survival was mainly observed in advanced cancer populations.^9^, ^10^

To the best of our knowledge, this is one of the largest study investigating the association between PROs and survival (particularly PFS) in patients with hematological cancers, with a patient population approximately five times larger than that of the previous publication on this topic by Jung et al.^13^ Moreover, unlike the Jung et al.^13^ study that created novel categories of “functional” and “symptom” scales by summarizing the function and symptom scales from the EORTC QLQ‐C30, respectively, the current study utilized the actual scales from the EORTC QLQ‐C30, as well as the additional key items (LYMS) from the FACT–Lym, to determine which scale(s) in particular were prognostic. This allowed for a greater understanding of which specific scales were most relevant.

The EORTC QLQ‐C30 physical functioning scale was the only PRO domain statistically significant both for OS and PFS while not only adjusting for IPI, but also for other key additional prognostic variables. This scale includes items that assess difficulty engaging in strenuous activities, taking long and short walks, and needing assistance with activities of daily living, or staying in a bed or chair during the day. Higher scores on each of these items represents poorer function, and once recoded into a transformed score, patients with lower scores on the physical functioning scale have poorer physical functioning. The fact that this scale retained significance in multivariable testing speaks to the relevance and importance of these questions in a DLBCL population and suggests that if clinicians are looking for one scale that can help with prognosis, this would be the one to use. Moreover, the significance of all four scales suggests that PROs may be as important as other more objective measures, such as laboratory values and stage, in identifying patients at risk.

What are the implications of the study findings for routine clinical practice? In looking at the results, two PRO scales in particular demonstrated consistent prognostic abilities at baseline, that is, the EORTC QLQ‐C30 physical functioning and the FACT–Lym LYMS. Both provide valuable and complementary information on the impact of the disease burden from the unique patient's viewpoint. However, further analyses are needed to provide clinicians with a pragmatic PRO‐based tool with clinically relevant cutoff points to identify patients at high risk of treatment failure or early relapse.

This study has several limitations. The main limitation is the exploratory nature of the analysis. Although this work was informed by literature that has increasingly demonstrated that PROs are prognostic for survival outcomes, the fact that there has been much less research conducted in patients with DLBCL meant that we could not take a confirmatory approach to our analyses. Future research with clinical trials of DLBCL patients utilizing the same measures may be used for confirmatory analyses. Also, further work is needed to possibly incorporate PRO scales into the IPI classification and develop a PRO‐based prognostic tool to aid in clinical decision‐making. However, our current findings lay the groundwork for additional research in this direction. Finally, given that our results are based on patients in a clinical trial setting, it is possible that our patients may not be fully representative of the larger DLBCL population typically seen in routine practice.

One of the key strengths of our study is the utilization of high‐quality data from the large GOYA dataset, which to the best of our knowledge, is one of the most extensive clinical trial datasets used to date to examine the prognostic value of PROs in a specific hematologic cancer population.^9^, ^12^ Additionally, we examined the prognostic value for PFS in addition to OS. Another strength was that the four scales were selected by univariate analysis as opposed to a reliance on the literature, hence, the scales were highly relevant to the current DLBCL population. Finally, to the best of our knowledge, this is the largest analyses of PROs as prognostic factors in patients with DLBCL.

In conclusion, the current study showed that in DLBCL, PROs provide independent prognostic information beyond a well‐established clinical index (IPI), suggesting that routine collection of PRO data in the setting of DLBCL could be used to identify individuals who are at greater risk of treatment failure or early relapse following first‐line immunochemotherapy. Also, this study may lay the groundwork for the future development of a “PRO‐based IPI prognostic tool” for patients with more advanced DLBCL. As observed in patients with other advanced hematologic malignancies, the integration of PROs into well‐established disease‐related prognostic indices may enhance accuracy of survival prediction.^23^ However, development of such a tool for patients with DLBCL would require further analyses and validation efforts also using external datasets.

## CONFLICT OF INTEREST

H. H., A. D., M. F., A. K., T. N., A. B., J. N. P., P. C. T.: Employment by F. Hoffmann‐La Roche Ltd. F. E.: personal fees from Amgen, Abbvie, Janssen, Takeda, and Novartis. Research support (to his Institution) from Abbvie, Amgen, Novartis, all outside the submitted work.

## AUTHOR CONTRIBUTIONS

Conception and design: Huang Huang, Asim Datye, Ming Fan, Tina Nielsen, Joseph N. Paulson, Peter C. Trask, and Fabio Efficace. Collection and assembly of data: Andrea Knapp, Tina Nielsen, and Joseph N. Paulson. Data analysis and interpretation: Peter C. Trask, Tina Nielsen, Joseph N. Paulson, Alessia Bottos, Huang Huang, Asim Datye, Ming Fan, and Fabio Efficace. Manuscript writing, final approval of manuscript, and accountable for all aspects of the work: all authors.

## Supporting information


Table S1
Figure S1
Figure S2
Figure S3
Figure S4

## Data Availability

Qualified researchers may request access to individual patient level data through the clinical study data request platform (https://vivli.org/). Further details on Roche's criteria for eligible studies are available here (https://vivli.org/members/ourmembers/). For further details on Roche's Global Policy on the Sharing of Clinical Information and how to request access to related clinical study documents, see here (https://www.roche.com/research_and_development/who_we_are_how_we_work/clinical_trials/our_commitment_to_data_sharing.htm).
