# Supplementary material for: Patient‐reported outcomes provide prognostic information for survival in patients with diffuse large B‐cell lymphoma: Analysis of 1239 patients from the GOYA study
Source: Cancer Med. 2022 Mar 23;11(17):3312–22. doi: 10.1002/cam4.4692 (PMC9468432; doi:10.1002/cam4.4692)
Supplement: Supplementary file 1 — Table S1 Figure S1 Figure S2 Figure S3 Figure S4 [file CAM4-11-3312-s001.docx]

**SUPPLEMENTARY MATERIAL**

**Supplementary Methods**

Eligible patients were aged ≥18 years with previously untreated, histologically documented, CD20-positive DLBCL and at least one bi-dimensionally measurable lesion (>1.5 cm in its largest dimension on computed tomography scan); ECOG PS 0–2; adequate organ function; and an IPI score of ≥2 (high, high-intermediate or low-intermediate risk). Patients with an IPI score of 1 (low risk) and aged ≤60 years, with or without bulky disease, and those with an IPI score of 0 with bulky disease (i.e. one lesion ≥7.5 cm) were also included.

Patients were treated with eight 21-day cycles of obinutuzumab 1000 mg (days 1, 8 and 15, cycle 1; day 1, cycles 2–8) or rituximab 375 mg/m^2^ (day 1, cycles 1–8) plus 6–8 cycles of CHOP. Cell-of-origin classification was based on gene expression profiling using the NanoString Lymphoma Subtyping Research-Use-Only assay (NanoString Technologies, Inc., Seattle, WA, USA).^1^ *BCL2* mutation status was defined with the FoundationOne Heme^TM^ platform (Foundation Medicine Incorporated [FMI], MA, USA) using genomic DNA extracted from diagnostic formalin-fixed, paraffin-embedded tissue sections. Total metabolic tumor volume was determined from baseline positron emission tomography images using an automated workflow program (MIM Software Inc, OH, USA).

**Supplementary Table 1.** Univariate analyses of common comorbidities in the GOYA study and their association with PFS and OS

| **Comorbidity (Y vs. N)** | **PFS**  **HR (95% CI)** | **OS**  **HR (95% CI)** |
| --- | --- | --- |
| Hypertension | 1.1 (0.9, 1.3) | 1.1 (0.9, 1.5) |
| Diabetes mellitus | 1.2 (0.9, 1.7) | 1.4 (0.96, 2.1) |
| Constipation | 1.0 (0.7, 1.4) | 0.8 (0.5, 1.3) |

**Supplementary Figure 1.** Univariate analysis of the prognostic value of a 10-point change in PRO scales for: (A) OS and (B) PFS. The four highest-ranking scales identified for both efficacy outcomes are shown in bold.


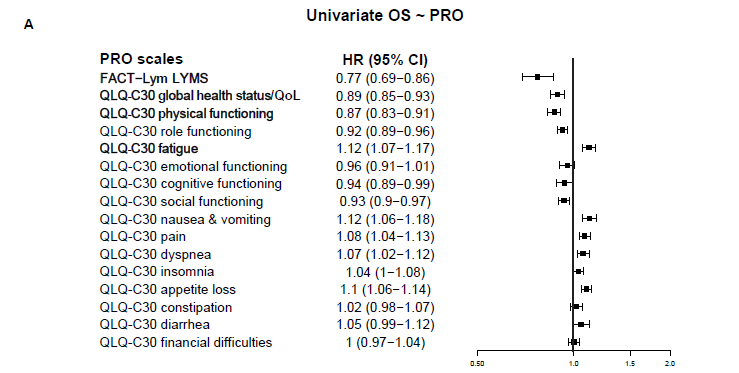


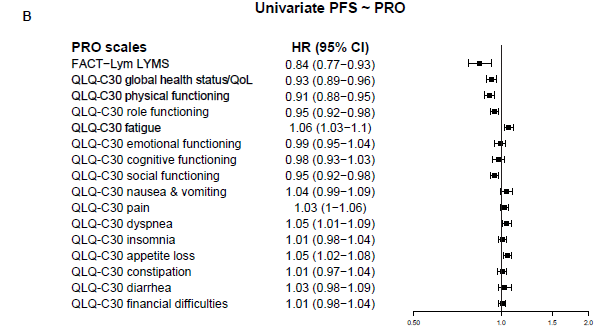


A higher score for the FACT–Lym LYMS and QLC-C30 functioning scales and global health status/QoL denotes a better level of functioning (i.e. a better state of the patient), while higher scores on the symptom scales indicate greater symptom severity. CI, confidence interval; FACT–Lym LYMS, Functional Assessment of Chronic illness Therapy-Lymphoma lymphoma-specific subscale; QoL, quality of life; HR, hazard ratio; OS, overall survival; PFS, progression-free survival; PRO, patient-reported outcome; QLQ-C30, European Organization for Research and Treatment of Cancer Quality of Life, Core 30.

**Supplementary Figure 2.** Sensitivity analysis for OS and PFS for: (A, B) patients with complete PRO data or missing data for at least one PRO scale, and (C, D) patients with missing data for all four PRO scales or missing data for ≤3 scales. Differences between groups were not statistically significant.


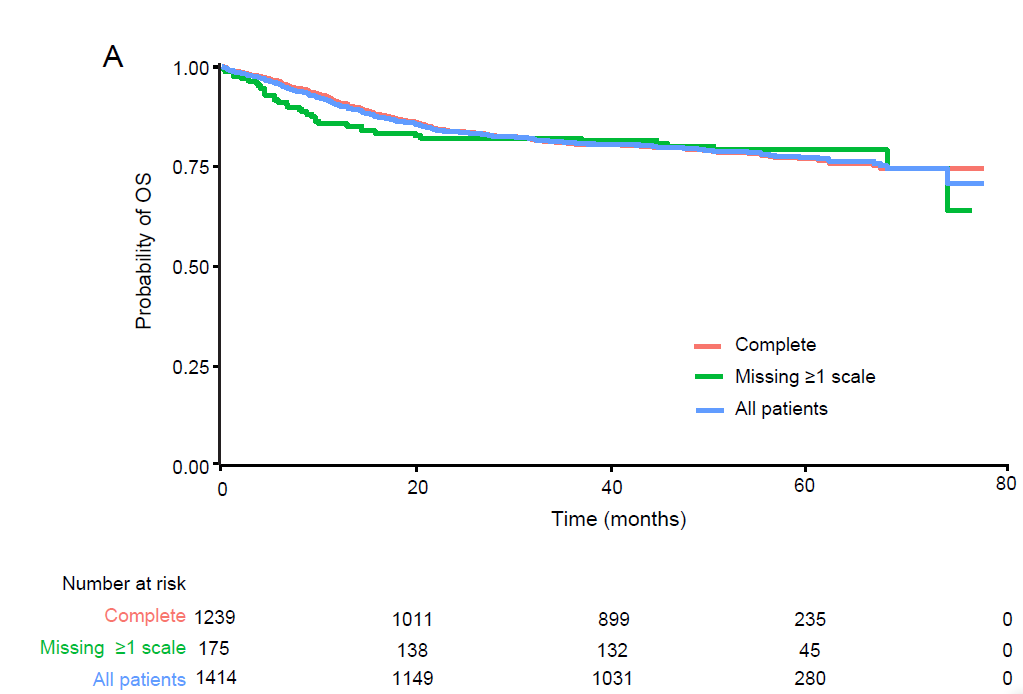


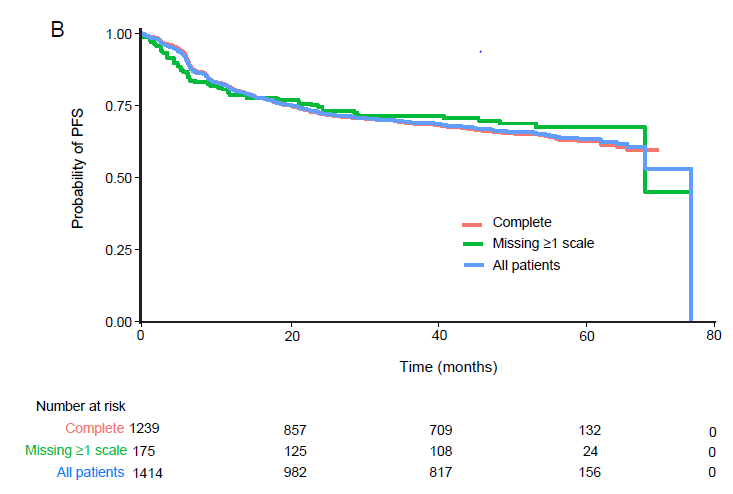

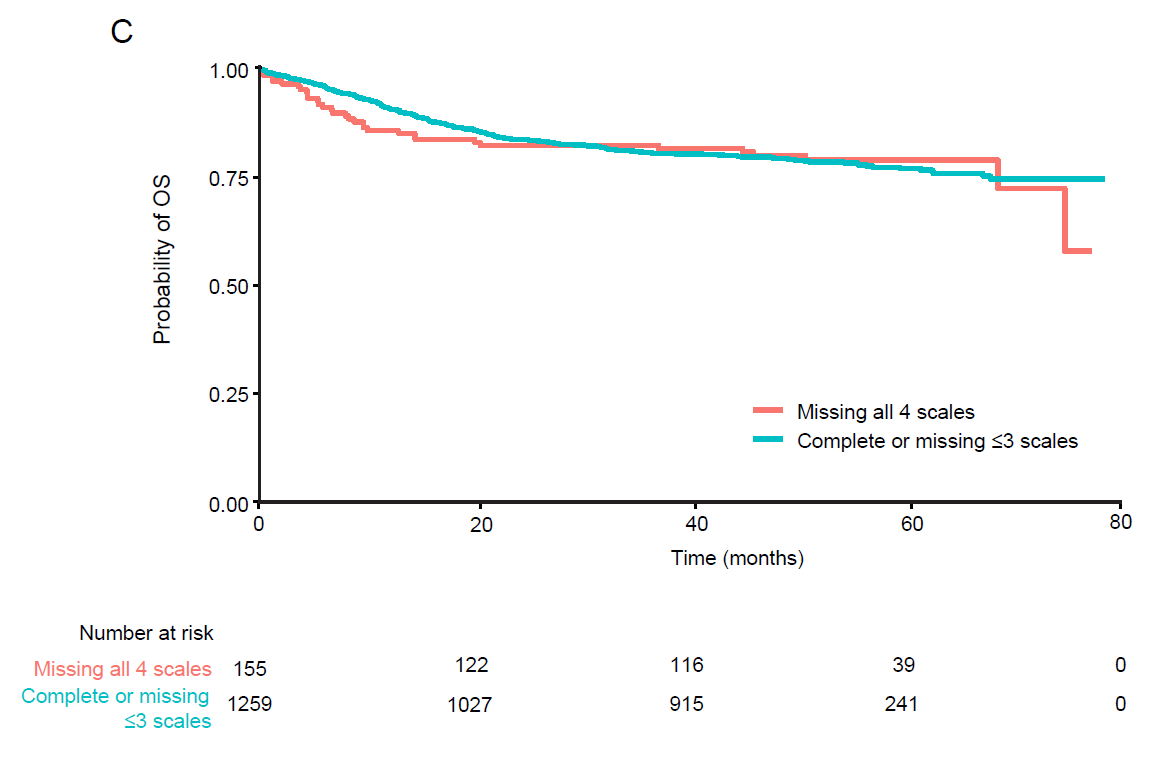

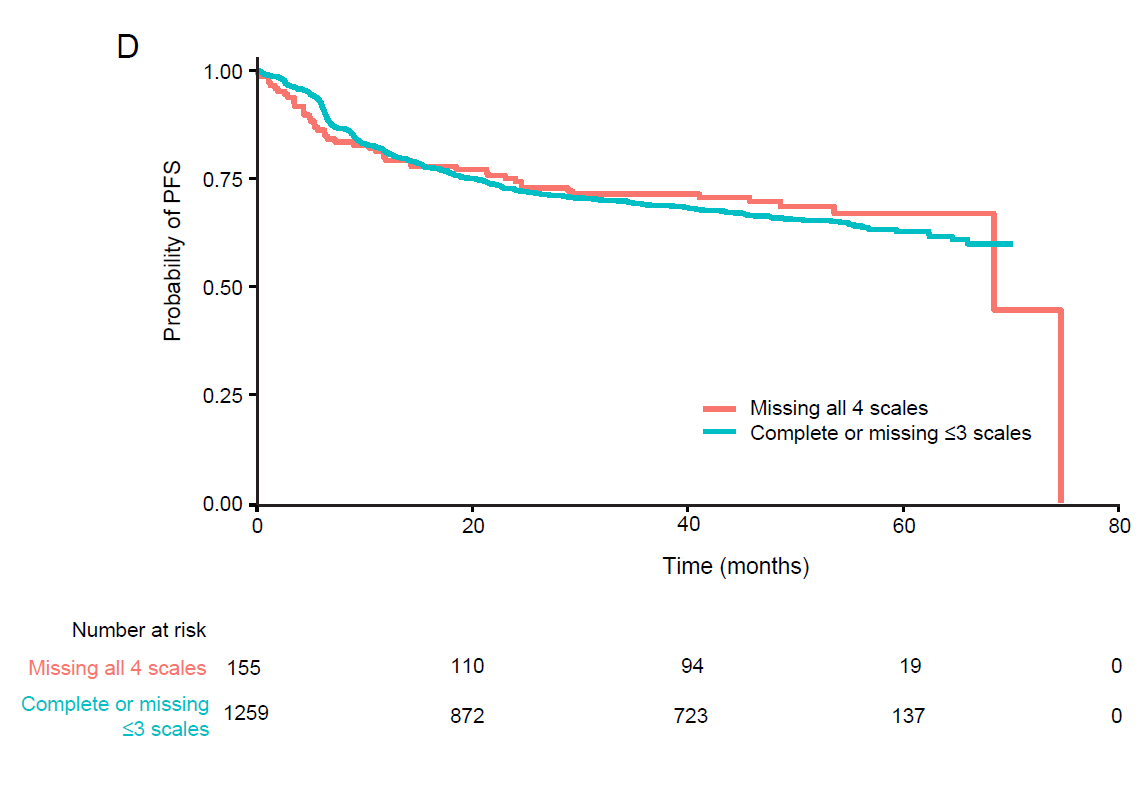


Scales: FACT–Lym LYMS, QLQ-C30 physical functioning, fatigue, global health status/QoL.

FACT-Lym LYMS, Functional Assessment of Chronic illness Therapy-Lymphoma lymphoma-specific subscale; QoL, quality of life; OS, overall survival; PFS, progression-free survival; PRO, patient-reported outcome; QLQ-C30, European Organization for Research and Treatment of Cancer Quality of Life, Core 30

**Supplementary Figure 3.** Sensitivity analyses for OS (A) and PFS (B): Cox regression analysis of the PRO scales after adjustment for: IPI, cell of origin, BCL2, total metabolic tumor volume, and the number of comorbidities^†^

**A**


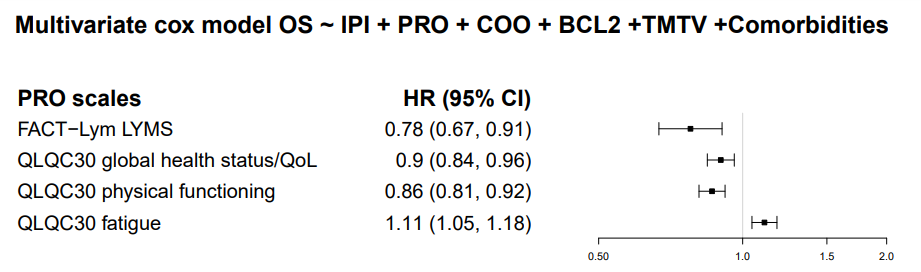


**B**


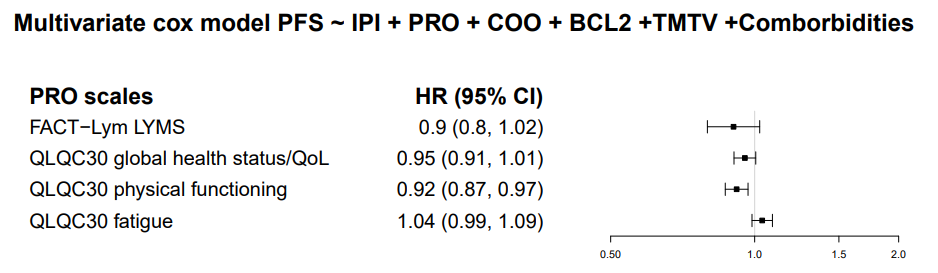


All four PRO scales were statistically significant in both Cox models. Higher scores for all four scales except the QLQ-C30 fatigue scale indicate better QoL or functioning, while higher scores on the QLQ-C30 fatigue scale indicate greater symptom severity.
^†^Adjustment for IPI (high/high-intermediate and low/low-intermediate), COO (ABC, GCB, unclassified, missing), *BCL2* status (positive, negative, missing), TMTV (continuous, mean imputed for missing), and the number of comorbidities.

ABC, activated B-cell like (subgroup); CI, confidence interval; COO, cell of origin; FACT–Lym LYMS, Functional Assessment of Chronic illness Therapy-Lymphoma lymphoma-specific subscale; GCB, germinal-center B cell-like (subgroup); HR, hazard ratio; QoL, quality of life; IPI, International Prognostic Index; PRO, patient-reported outcome; OS, overall survival; QLQ-C30, European Organization for Research and Treatment of Cancer Quality of Life, Core 30; TMTV, total metabolic tumor volume

**Supplementary Figure 4.** TMTV by the four PRO scales, separated by high and low health-related quality of life (HR QoL) defined by the median of each PRO scale.


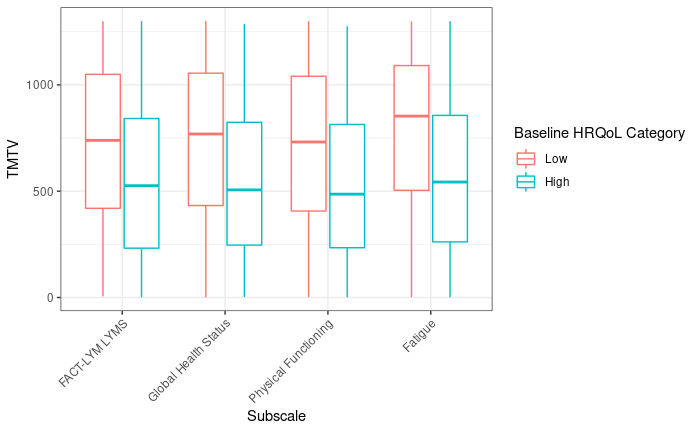


**References**

1. Bolen C, Klanova M, Trnèny M: Prognostic impact of somatic mutations in diffuse large B-cell lymphoma and relationship to cell-of-origin: data from the phase III GOYA study. Haematologica 9:2298-2307, 2020
